# Clinical characteristics and prognostic factors of Hurthle cell carcinoma: a population based study

**DOI:** 10.1186/s12885-020-06915-0

**Published:** 2020-05-12

**Authors:** Xingtong Zhou, Zhibo Zheng, Chuyan Chen, Bangbo Zhao, Hongtao Cao, Tianhao Li, Xudong Liu, Weibin Wang, Yongning Li

**Affiliations:** 1 Department of Breast Surgery, Peking Union Medical College Hospital, Chinese Academy of Medical Sciences, Beijing, China; 2 Department of International Medical Services, Peking Union Medical College Hospital, Chinese Academy of Medical Sciences, Beijing, China; 3 Peking Union Medical College, Chinese Academy of Medical Sciences, Beijing, China; 4 Department of General Surgery, Peking Union Medical College Hospital, Chinese Academy of Medical Sciences, No.1 Shuaifuyuan Wangfujing Dongcheng District, Beijing, China; 5 Medical Science Research Center, Peking Union Medical College Hospital, Chinese Academy of Medical Sciences, Beijing, China

**Keywords:** Thyroid Hurthle cell carcinoma, SEER database, Survival factors

## Abstract

**Background:**

Thyroid Hurthle cell carcinoma (HCC) is a rare disease with high risk of invasion and metastasis and poor prognosis. The clinical characteristics, prognosis and treatment of HCC are still controversial, and clinical data are still limited to some case reports. Therefore, understanding the characteristics and survival factors of HCC is clinically necessary.

**Methods:**

This study collected data from HCC patients diagnosed pathologically from 2004 to 2015, including basic population characteristics, tumor characteristics, and epidemiological and survival data. The data were extracted from the Surveillance, Epidemiology, and End Results (SEER) database to conduct a population cohort study.

**Results:**

A total of 2101 HCC patients with an average age of 55.42 ± 15.27 years were enrolled in this study. Of them, 1740 (82.82%) patients had local disease, 245 (11.66%) had regional disease, and 89 (4.24%) had distant disease. Total thyroidectomy was performed in 1669 (79.44%) patients, partial thyroidectomy was performed in 382 (18.18%) patients, and radioactive iodine (RAI) was used in 1155 (54.97%) patients. The 5-year and 10-year cancer-specific survival rate was 95.4 and 92.6%, respectively. The distant disease group had significantly more male patients, multifocal tumors, and extensive tumors compared to the local disease group. Multivariate survival analysis showed that age (*P* < 0.05), SEER stage (*P* < 0.001), and T-stage (*P* = 0.001) had significant effects on survival. There was no significant difference in survival between total and partial thyroidectomy (*P* = 0.078), or between RAI and non-RAI (*P* = 0.733).

**Conclusion:**

Male gender, multifocal tumors, and extended tumors are associated with increased risk of late stage HCC. Age over 45 years, distant SEER stage, and late T-stage are independent risk factors for mortality in HCC.

## Background

Oxyphilic cells, also known as Hurthle cells, are present in some thyroid tumors and nontumor tissues, such as thyroiditis and nodular goiter [1]. Thyroid oxyphilic tumors, also known as Hurthle cell tumors, refer to those thyroid tumors that entirely or predominantly (> 75%) consist of oxyphilic thyroid follicular cells [2]. Thyroid oxyphilic tumors have been identified by the World Health Organization as a special type of tumor of the thyroid follicles that are distinguished from thyroid follicular tumors [3]. Thyroid oxyphilic tumors can be benign (Hurthle cell adenoma) or malignant (Hurthle cell carcinoma, HCC). HCC is characterized by capsule invasion and/or vascular invasion. Hurthle cell adenocarcinoma is a rare invasive thyroid malignancy, accounting for 3 to 4% of all thyroid malignancies.

Compared to differentiated thyroid carcinoma, HCC has high risk of lymph node metastasis and distant metastasis and is less sensitive to radioiodine therapy [4, 5]. HCC is a rare disease with unique pathological characteristics and biological behaviors. There is still no consensus on its best surgical treatment method. Our study aimed to find out the characteristics and survival factors of HCC by analyzing patient data from the Surveillance, Epidemiology, and End Results (SEER) database.

## Methods

### Data collection

All patients diagnosed with oxyphilic adenocarcinoma between 2004 and 2015 according to the International Classification of Disease were identified in the SEER database. Data of patient demographics, surgeries, postoperative treatments, tumor pathology, SEER stage, and disease-specific survival were collected. The SEER stage was used for tumor staging [6].

### Statistical analysis

Demographic, tumor features, and treatment methods were summarized with descriptive statistics. Continuous data are presented as means and standard deviations. Categorical data are presented as counts or percentages. Comparisons of the continuous data were made using the one-way ANOVA test followed by the Tukey’s post-hoc test for between three groups or using the independent student t-test for between two groups. Univariate and multivariate Cox proportional hazard models were used to assess the relative impacts of risk factors for HCC. Kaplan-Meier survival curves were constructed for cancer-specific mortality, while the differences between the curves were tested by the log-rank test. All statistical analyses were conducted with SPSS 25.0 (SPSS Inc., Chicago, IL, USA) or GraphPad Prism 7 (GraphPad Software, CA, USA). *P* < 0.05 was considered statistically significant.

## Results

### Patient characteristics

A total of 3084 HCC patients were identified in the database, accounting for 2.4% of all differentiated thyroid carcinoma and 6.67% of follicular thyroid carcinoma patients in the same period. Among them, 2101 patients had comprehensive detailed information and were included in the analysis. Patients were divided into three groups according to the SEER stage (local, regional, and distant). The patient characteristics are listed in Table 1.

**Table 1:** Patient characteristics

Patient number, n	2101
Age, year	55.42 ± 15.27
Gender, n (%)	
Male	606 (28.84)
Female	1495 (71.16)
Age, year	
< 45	508 (24.18)
≥ 45	1593 (75.82)
Race, n (%)	
White	1736 (82.63)
Black	188 (8.95)
American Indian/Alaska Native, Asian/Pacific Islander	144 (6.85)
Unknown	33 (1.57)
SEER stage^a^, n (%)	
Local	1740 (82.82)
Regional	245 (11.66)
Distant	89 (4.24)
Unspecified	27 (1.28)
Lymph node examination, n (%)	
Not examined	1406 (66.92)
Negative	574 (27.32)
Positive	92 (4.38)
Unspecified	29 (1.38)
Tumor grade, n (%)	
Well differentiated	282 (13.42)
Moderately differentiated	80 (3.81)
Poorly differentiated	41 (1.95)
Undifferentiated	13 (0.62)
Unspecified	1685 (80.20)
Tumor multifocality^b^, n (%)	
No	1657 (78.87)
Yes	352 (16.75)
Unspecified	92 (4.38)
Tumor extension, n (%)	
Intrathyroidal	1768 (84.15)
Extrathyroidal	301 (14.33)
Unspecified	32 (1.52)
T-stage, n (%)	
T1	486 (23.13)
T2	707 (33.65)
T3	708 (33.70)
T4	98 (4.66)
Unspecified	102 (4.85)
N-stage, n (%)	
N0	1913 (91.05)
N1	113 (5.38)
Unspecified	75 (3.57)
M-stage, n (%)	
M0	1995 (94.95)
M1	59 (2.81)
Unspecified	47 (2.24)
Surgery, n (%)	
No	29 (1.38)
Total thyroidectomy	1669 (79.44)
Partial thyroidectomy	382 (18.18)
Unspecified	21 (0.99)
Radiotherapy, n (%)	
No	850 (40.46)
Radioactive iodine	1155 (54.97)
Unspecified	96 (4.57)

SEER, Surveillance, Epidemiology, and End Results

^a^The local stage includes localized disease only. The regional stage includes regional disease by direct extension only, regional lymph nodes only, and regional disease by both direct extension and lymph node involvement. The distant stage includes disease that involve distant sites and/or lymph nodes

^b^The tumor has multiple centers, and the foci are not contiguous

### Comparison of patients with different SEER stages

There was significant difference in age between patients with different SEER stages (*P* < 0.001; Table 2). The distant group had significantly more patients with male sex (*P* = 0.001), tumor multifocality (P < 0.001), and tumor extension (P < 0.001) compared to the local group (P < 0.001; Table 2). We also found that the TNM stage was consistent with the SEER stage (Table 2).

**Table 2:** Patients with different SEER stages

Characteristics	Local disease (*n* = 1740)	Regional disease (*n* = 245)	Distant disease (*n* = 89)	*P*-value	P^a^-value	P^b^-value	P^c^-value
Age (year)	54 ± 14.8	61 ± 17.1	69 ± 13.5	< 0.001	< 0.001	< 0.001	< 0.001
Gender, n (%)				0.001	0.010	0.370	0.005
Male	476 (27.4)	87 (35.5)	37 (41.6)				
Female	1264 (72.6)	158 (64.5)	52 (58.4)				
Race, n (%)				0.016	0.003	0.064	0.898
White	1435 (82.5)	206 (84.1)	72 (82.6)				
Black	168 (9.7)	9 (3.7)	10 (11.2)				
American Indian/Alaska Native, Asian/Pacific Islander	111 (6.4)	25 (10.2)	7 (7.9)				
Unknown	26 (1.5)	5 (2.0)	0				
Tumor multifocality, n (%)				< 0.001	< 0.001	< 0.001	< 0.001
No	1428 (82.1)	173 (70.6)	51 (57.3)				
Yes	268 (15.4)	58 (23.7)	25 (28.1)				
Unspecified	44 (2.5)	14 (5.7)	13 (14.6)				
Tumor extension, n (%)				< 0.001	< 0.001	0.023	< 0.001
Intrathyroidal	1700 (97.7)	49 (20.0)	19 (21.3)				
Extrathyroidal	40 (2.3)	195 (79.6)	66 (74.2)				
Unspecified	0	1 (0.4)	4 (4.5)				
T-stage, n (%)				< 0.001	< 0.001	< 0.001	< 0.001
T1	467 (26.8)	16 (6.5)	3 (3.4)				
T2	678 (39.0)	21 (8.6)	8 (9.0)				
T3	526 (30.2)	164 (66.9)	18 (20.2)				
T4	1 (0.1)	42 (17.1)	54 (60.7)				
Unspecified	68 (3.9)	2 (0.8)	6 (6.7)				
N-stage, n (%)				< 0.001	< 0.001	0.023	< 0.001
N0	1703 (97.9)	153 (62.4)	52 (58.4)				
N1	0	85 (34.7)	28 (31.5)				
Unspecified	37 (2.1)	7 (2.9)	9 (10.1)				
M-stage, n (%)				< 0.001	0.167	< 0.001	< 0.001
M0	1718 (98.7)	240 (98.0)	30 (33.7)				
M1	0	0	59 (66.3)				
Unspecified	22 (1.3)	5 (2.0)	0				

*SEER* Surveillance, Epidemiology, and End Results. P^a^, local stage vs. regional stage; P^b^, regional stage vs. distant stage; P^c^, local stage vs. distant stage

### Comparison of patients with different surgical procedures

A total of 2051 patients were surgically managed, including 1669 (79.44%) cases of total thyroidectomy and 382 (18.18%) cases of partial thyroidectomy (Table 3). Among the patients undergoing total thyroidectomy, 61.8% were married, versus 54.5% of married patients in the partial thyroidectomy group (*P* = 0.027). There were significantly more patients with T3, T4, N1, and M1 stages in the total thyroidectomy group than in the partial thyroidectomy group.

**Table 3:** Patients with different surgical procedures

Characteristics	Total thyroidectomy (*n* = 1669)	Partial thyroidectomy (*n* = 382)	P-value
Age	55 ± 15.0	55 ± 16.5	0.136
Gender, (%)			0.556
Male	484 (29.0)	105 (27.5)	
Female	1185 (71.0)	277 (72.5)	
Race, n (%)			0.667
White	1387 (83.1)	309 (80.9)	
Black	148 (8.9)	36 (9.4)	
American Indian/Alaska Native, Asian/Pacific Islander	113 (6.8)	30 (7.9)	
Unknown	21 (1.3)	7 (1.8)	
SEER stage, n (%)			0.023
Local	1388 (83.2)	340 (89.0)	
Regional	208 (12.5)	35 (9.2)	
Distant	68 (4.1)	6 (1.6)	
Unspecified	5 (0.3)	1 (0.3)	
Tumor multifocality, n (%)			0.072
No	1324 (79.3)	317 (83.0)	
Yes	295 (17.7)	50 (13.1)	
Unspecified	50 (3.0)	15 (3.9)	
Tumor extension, n (%)			0.394
Intrathyroidal	1420 (85.1)	335 (87.7)	
Extrathyroidal	241 (14.4)	46 (12.0)	
Unspecified	8 (0.5)	1 (0.3)	
T-stage, n (%)			0.022
T1	391 (23.4)	93 (24.3)	
T2	566 (33.9)	137 (35.9)	
T3	577 (34.6)	122 (31.9)	
T4	80 (4.8)	8 (2.1)	
Unspecified	55 (3.3)	22 (5.8)	
N-stage, n (%)			< 0.001
N0	1535 (92.0)	361 (94.5)	
N1	99 (5.9)	6 (1.6)	
Unspecified	35 (2.1)	15 (3.9)	
M-stage, n (%)			0.005
M0	1605 (96.2)	368 (96.3)	
M1	44 (2.6)	3 (0.8)	
Unspecified	20 (1.2)	11 (2.9)	
Radiotherapy, n (%)			< 0.001
No	557 (33.4)	262 (68.6)	
Radioactive iodine	1033 (61.9)	111 (29.1)	
Unspecified	79 (4.7)	9 (2.4)	

SEER: Surveillance Epidemiology and End Results

### Patient survival

The 5-year and 10-year cancer-specific survival (CSS) rates were 95.4 and 92.6%, respectively. Univariate survival analysis showed that age > 45 years, late SEER stage, tumor extension, lymph node metastasis, and late TNM stage were associated with poor prognosis (all *P* < 0.05). Female patients and surgically treated patients had significantly longer survival time (*P* < 0.001). Multivariate analysis was used to identify the independent prognostic factors. The CSS was 134.36 ± 1.71 months for patients over 45 years of age, and 141.59 ± 1.23 months for patients under 45 years of age, suggesting that age ≥ 45 was an independent prognostic factor (hazard ratio [HR] = 3.595, 95% confidence interval: 1.415–9.131). In addition, regional disease, distant disease, T3 stage, and T4 stage were also independent prognostic factors (HR > 1) (Table 4).

**Table 4:** Univariate and multivariate Cox analyses of clinical features for cancer-related survival rates

Features	Univariate		Multivariate	
	HR (95% CI)	*P*-value	HR (95% CI)	*P*-value
Gender				
Male	1		1	
Female	0.415 (0.278–0.619)	< 0.001	0.614 (0.409–0.924)	0.019
Age (year)				
< 45	1		1	
≥ 45	6.354 (2.582–15.635)	< 0.001	4.204 (1.695–10.432)	0.002
Race				
White	1		–	–
Black	0.960 (0.465–1.984)	0.913	–	–
Other	0.646 (0.237–1.761)	0.393	–	–
SEER stage				
Local	1		1	
Regional	8.454 (4.823–14.817)	< 0.001	8.015 (4.555–14.101)	< 0.001
Distant	61.625 (3.772–31.568)	< 0.001	46.219 (27.108–78.804)	< 0.001
Tumor multifocality				
No	1		–	–
Yes	1.334 (0.808–2.202)	0.260	–	–
Tumor extension				
Intrathyroidal	1		–	–
Extrathyroidal	12.342 (8.062–18.892)	< 0.001	–	–
Lymph node examination				
Not examined	1		–	–
Negative	1.060 (0.646–1.741)	0.817	–	–
Positive	6.029 (3.494–10.403)	< 0.001	–	–
T-stage				
T1	1		–	–
T2	0.701 (0.226–2.173)	0.538	–	–
T3	4.866 (2.060–11.496)	< 0.001	–	–
T4	42.305 (17.874–100.132)	< 0.001	–	–
N-stage				
N0	1		–	–
N1	8.863 (5.561–14.124)	< 0.001	–	–
M-stage				
M0	1		–	–
M1	32.934 (21.214–51.129)	< 0.001	–	–
Surgery				
No	1		1	
Total thyroidectomy	0.085 (0.044–0.166)	< 0.001	0.335 (0.152–0.741)	0.007
Partial thyroidectomy	0.046 (0.019–0.114)	< 0.001	0.266 (0.096–0.742)	0.011
Radiotherapy				
No	1		–	–
Yes	0.606 (0.405–0.905)	0.014	–	–

*CI* confidence interval, *SEER* Surveillance Epidemiology and End Results

**Fig. 1:**
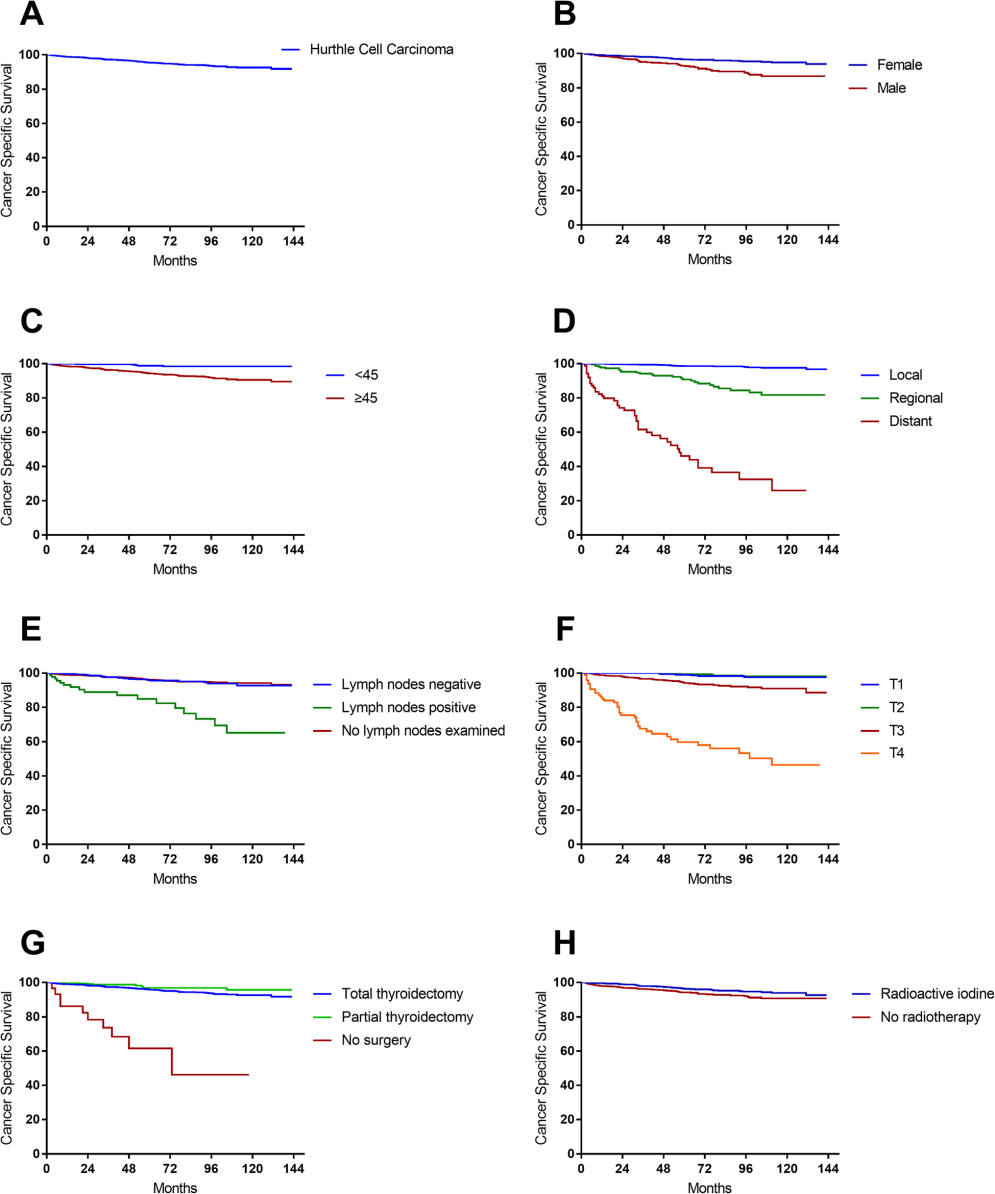
Survival analysis. (**a**) Cancer-specific survival of the patients. (**b**) Cancer-specific survival of patients under and over 45 years of age (*P* = 0.007). (**c**) Cancer-specific survival of patients with different SEER stages (*P* < 0.001). (**d**) Cancer-specific survival of patients with different lymph node status (*P* < 0.001). (**e**) Cancer-specific survival of patients with different T stages (*P* < 0.001). (**F**) Cancer-specific survival of surgically and non-surgically managed patients (*P* < 0.001)

Kaplan-Meier curves were constructed to describe the survival of different groups. Figure 1a shows the CSS of all HCC patients. The 5-year and 10-year survival rates were 96.53 and 94.77% for female patients, and 92.61 and 86.88% for male patients (Fig. 1b). The 5-year and 10-year survival rates were 98.85 and 98.48% for patients under 45 years of age, and 94.28 and 90.51% for patients over 45 years of age (Fig. 1c). The 5-year and 10-year survival rates were 98.6 and 97.6% for patients with local disease, and 90.8 and 81.7% for patients with regional disease. However, the 5-year and 10-year survival rates were only 46.0 and 26.0% for patients with distant disease (Fig. 1d). The 5-year and 10-year survival rates were 95.6 and 92.7% for patients with negative lymph nodes, which was significantly higher than the 84.9 and 65.3% for those with positive lymph nodes (Fig. 1e). The 10-year survival rates of T1 and T2 stage patients were 97.57 and 98.13%. The 5-year and 10-year survival rates of T3 stage patients was 94.23 and 90.93%, respectively, which were significantly higher than the 5-year and 10-year survival rates of 59.78 and 46.45% for the T4 patients (Fig. 1f). There was no significant difference in 10-year survival rate between patients treated with total thyroidectomy and those treated with partial thyroidectomy (92.6% vs. 95.6%, *P* = 0.078). The 5-year and 10-year survival rates were 61.6 and 46.2% for non-surgically managed patients (Fig. 1g). The 5-year and 10-year survival rates were 96.28 and 93.86% for patients treated with RAI, and 94.18 and 90.78% for patients without RAI (Fig. 1h).

## Discussion

Our study analyzed the basic characteristics, treatment methods, and survival of patients with HCC. From 2000 to 2015, 86 patients with thyroid Hurthle cell tumors were treated at the Peking Union Medical College Hospital, of which only 5 patients were diagnosed with thyroid HCC. Most literatures on HCC are case reports rather than clinical studies with large samples. Moreover, diagnosis of HCC relies on postoperative pathology. HCC is also characterized by multifocality with high risk of lymph node metastases and distant metastases [7]. Approximately 10–20% of patients have metastases when diagnosed with HCC. Up to 37% of extrathyroidal extension HCC metastasizes to the cervical lymph nodes [8]. There is still no consensus on the optimal treatment method for HCC, and the postoperative effect of radioactive iodine treatment is unclear.

The SEER database has been utilized to find the differences between HCC and other thyroid cancers [9–11]. Compared to other differentiated thyroid cancers, HCC is more aggressive with higher risk of distant metastasis and poor prognosis. For the first time, our study used the data of 2101 patients in the SEER database from 2004 to 2015 to describe the clinical characteristics of HCC patients and identify the prognostic factors of CSS.

HCC is more common in women with a male to female ratio of approximately 1:2 to 1:4 [1], which is similar to our result of the ratio of male to female of 1:2.47. The first symptoms of HCC patients may include thyroid nodules or cervical lymphadenectasis. The SEER staging plays an important role in the treatment and prognosis of HCC. Therefore, our study also analyzed the relationship between the HCC clinical characteristics and SEER staging. The SEER staging integrates clinical and pathological data to provide accurate evaluation of the degree of disease. Our study found that older age, male sex, multifocal tumors, and extensive tumors were risk factors of late SEER stages. These results suggest that HCC has similar characteristics with other differentiated thyroid carcinomas.

Surgery is still the most effective treatment for HCC [10, 12]. Our study found no significant difference in CSS between patients treated with total thyroidectomy and those treated with partial thyroidectomy. This result suggests that the cancer-specific prognosis of HCC is not greatly affected by surgical methods. We speculate that partial thyroidectomy is sufficient for single, small tumors without extrathyroidal invasion. Compared with total thyroidectomy, partial thyroidectomy has fewer complications and less intraoperative injury, but has comparable survival time.

Radioactive iodine (RAI) is widely used for the treatment of differentiated thyroid cancer, especially papilla thyroid carcinoma. However, HCC is insensitive to RAI due to the low iodine uptake rate. Despite this, RAI is used in some patients after total thyroidectomy [13]. The multivariate Cox analysis found that RAI treatment did not significantly improve the prognosis of HCC. However, the univariate Cox analysis showed that HCC patients can benefit from RAI treatment, which is consistent with previous findings [12].

HCC is associated with a higher metastasis rate and a lower survival rate compared to other differentiated thyroid cancers [14]. HCC with distant metastases has a 5-year mortality rate of up to 80% [15]. Age, tumor size, and sex are prognostic factors of HCC, and tumor extension and recurrence often indicate poor prognosis and increased mortality [16]. The reported 5-year and 10-year survival rates for nonmetastatic HCC are 85.1 and 71.1%, respectively [17]. In our study, the 5-year and 10-year CSS rate for HCC were 95.4 and 92.6%, respectively. The higher survival rates in our study may be explained by the advancement in diagnosis and treatment of HCC in the last decade.

Our study has limitations. First, a small portion of the included patients had no data of race or tumor characteristics. For example, 80.2% of the patients had unclear tumor grades, and 2–5% had unclear TNM stages. Therefore, the SEER stage was adopted to describe the disease progression rather than the TNM stage. Second, the tumor grade data was excluded from the analysis due to incomplete information. Third, the SEER database has no detailed information on tumor multifocality, tumor extension, and completion thyroidectomy.

## Conclusions

The SEER stage is an independent prognostic factor for HCC, and distant disease is associated with significantly poor prognosis. Age over 45 years, distant SEER stage, and late T-stage are independent risk factors for mortality in HCC. There was no significant difference in survival between patients treated with partial thyroidectomy and those treated with total thyroidectomy. RAI treatment may possibly improve patient survival after thyroidectomy.

## Abbreviations

HCC: Hurthle cell carcinoma
SEER: Surveillance, Epidemiology, and End Results
